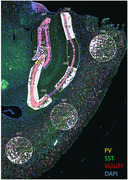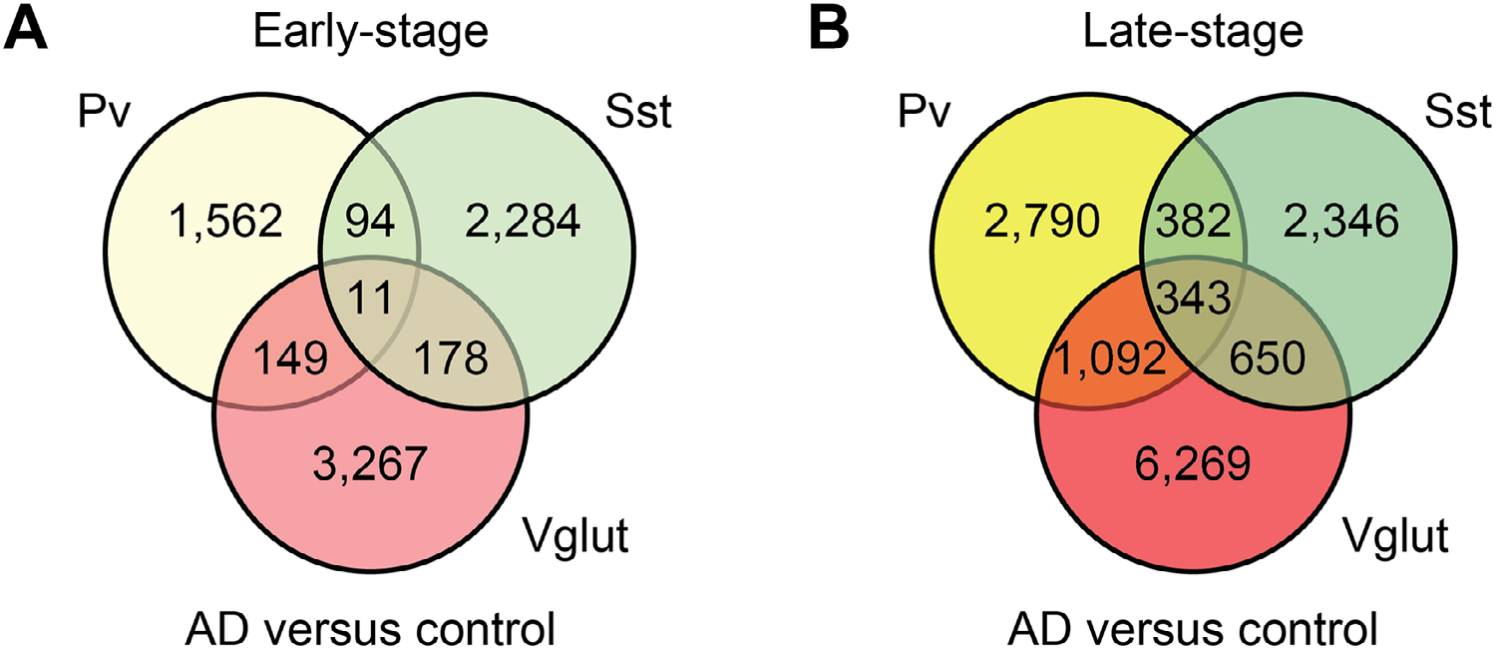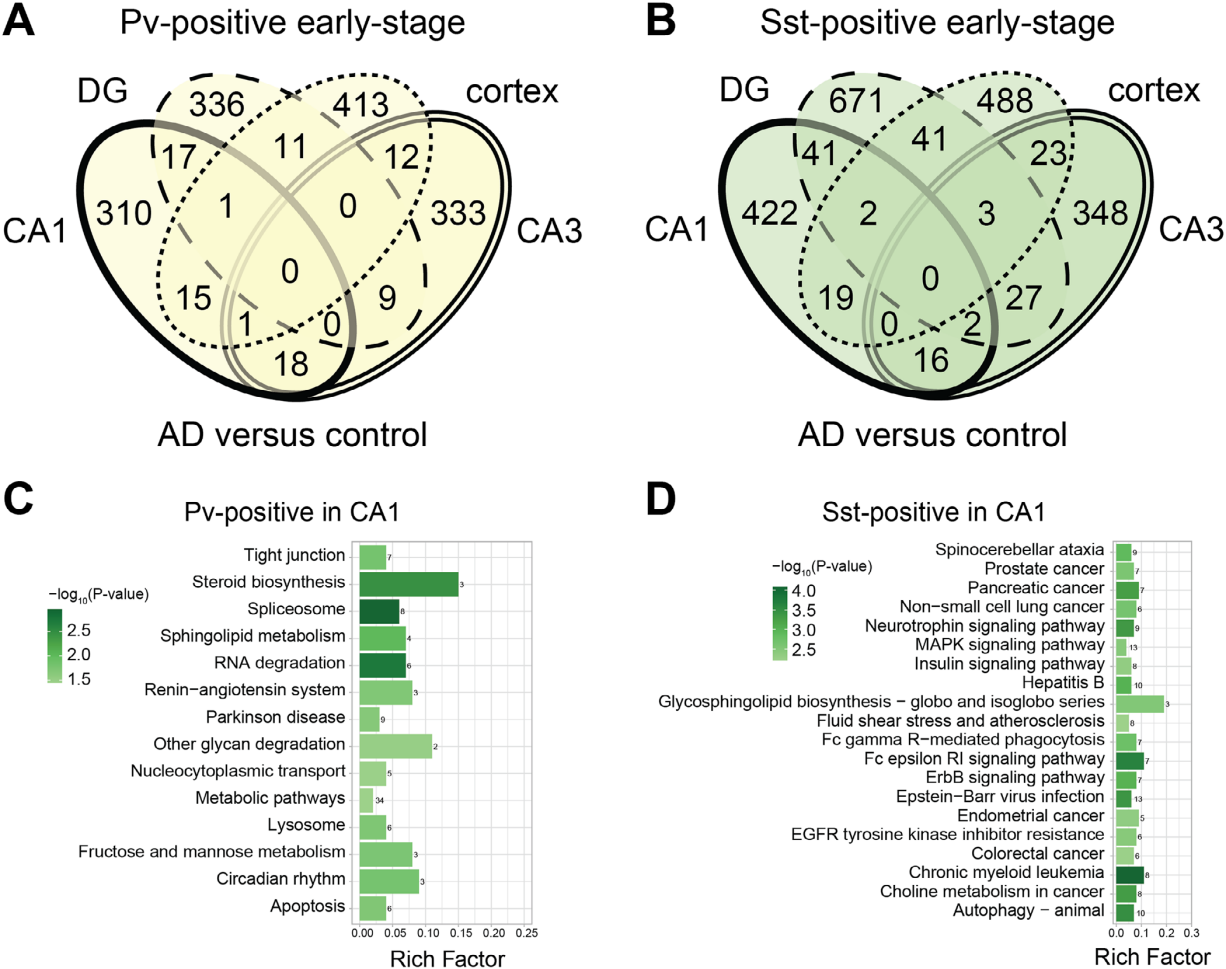# Pathways of inhibitory interneuron dysregulation in 5XFAD mice

**DOI:** 10.1002/alz.086555

**Published:** 2025-01-03

**Authors:** Kevin S Chen, Mohamed Noureldein, Diana M Rigan, John M Hayes, Masha G Savelieff, Eva L Feldman

**Affiliations:** ^1^ University of Michigan, Ann Arbor, MI USA; ^2^ University of North Dakota, Grand Forks, ND USA

## Abstract

**Background:**

Inhibitory interneurons normally regulate neural networks underlying memory and cognition, but are disrupted in Alzheimer’s disease. Proper interneuron activity reduces amyloid‐beta, whereas hyperexcitability elevates amyloid levels. Still, the underlying pathologic processes mediating interneuron dysfunction remain unknown. Therefore, we employed a spatial transcriptomics approach to map transcriptomic profiles of interneurons in a temporal and spatial manner.

**Method:**

Coronal hemibrain sections from early stage (12 wks) and late stage (30 wks) male mice (5XFAD) underwent fluorescence *in situ* hybridization to identify interneuron subtypes (parvalbumin‐expressing, PV+, or somatostatin‐expressing, SST+). Slides were submitted for GeoMx DSP spatial transcriptomics (2 duplicate slides per timepoint, n = 2 5XFAD and n = 2 WT per slide). Regions of interest (ROIs) were defined in hippocampal and cortical regions (Figure 1). UV‐liberated barcodes from each ROI, restricted to PV+ or SST+ interneurons, were collected by microcapillary and sequenced by the NanoString Max/Flex nCounter system. Differentially expressed genes (DEGs) were assessed by linear mixed‐effect model and the GeoMx analysis suite, and attribution to respective time points, cell type, and anatomic region were sequentially parsed.

**Result:**

In early‐stage disease, 1,562 DEGs were uniquely expressed by PV+ interneurons and 2,284 DEGs were uniquely expressed by SST+ interneurons. Similarly, at late‐stage disease, 2,790 DEGs were expressed by PV+ interneurons and 2,346 DEGs were uniquely expressed by SST+ interneurons (Figure 2). Focusing on the CA1 region, previously implicated as having a central role in hippocampal dysfunction, interneurons again showed unique alterations. Early‐stage PV+ interneurons in CA1 uniquely expressed 310 DEGs, with “metabolic pathways” having the most DEGs (Figure 3). By contrast, early‐stage SST+ interneurons in CA1 uniquely expressed 422 DEGs, enriched in pathways canonically linked to “amyotrophic lateral sclerosis” and “Alzheimer disease.”

**Conclusion:**

The most significant altered pathways within AD interneurons at the earliest stages of disease included neurodegeneration and metabolism pathways. Zero or few DEGs overlapped across all regions or neuronal subtypes. Thus, interneurons display distinct profiles and transcriptional changes by brain area at early‐ and late‐stage disease. These findings will inform future mechanistic experimentation, and suggest investigation into neural circuit dysfunction in AD will require consideration of anatomic subregion specificity.